# The paediatric Bohler's angle and crucial angle of Gissane: a case series

**DOI:** 10.1186/1749-799X-6-2

**Published:** 2011-01-10

**Authors:** Matthew J Boyle, Cameron G Walker, Haemish A Crawford

**Affiliations:** 1 Department of Paediatric Orthopaedics, Starship Children's Hospital, Park Road, Auckland, New Zealand; 2 Department of Engineering Science, The University of Auckland, Symonds Street, Auckland, New Zealand

## Abstract

**Background:**

Bohler's angle and the crucial angle of Gissane can be used to assess calcaneal fractures. While the normal adult values of these angles are widely known, the normal paediatric values have not yet been established. Our aim is to investigate Bohler's angle and the crucial angle of Gissane in a paediatric population and establish normal paediatric reference values.

**Method:**

We measured Bohler's angle and the crucial angle of Gissane using normal plain ankle radiographs of 763 patients from birth to 14 years of age completed over a five year period from July 2003 to June 2008.

**Results:**

In our paediatric study group, the mean Bohler's angle was 35.2 degrees and the mean crucial angle of Gissane was 111.3 degrees. In an adult comparison group, the mean Bohler's angle was 39.2 degrees and the mean crucial angle of Gissane was 113.8 degrees. The differences in Bohler's angle and the crucial angle of Gissane between these two groups were statistically significant.

**Conclusion:**

We have presented the normal values of Bohler's angle and the crucial angle of Gissane in a paediatric population. These values may provide a useful comparison to assist with the management of the paediatric calcaneal fracture.

## Background

Bohler's angle and the crucial angle of Gissane are commonly assessed when evaluating patients with calcaneal fractures. Traumatic alteration of these angles can be used as a measure of fracture severity, with one goal of surgical management being restoration of these angles to normal values.

**Figure 1:**
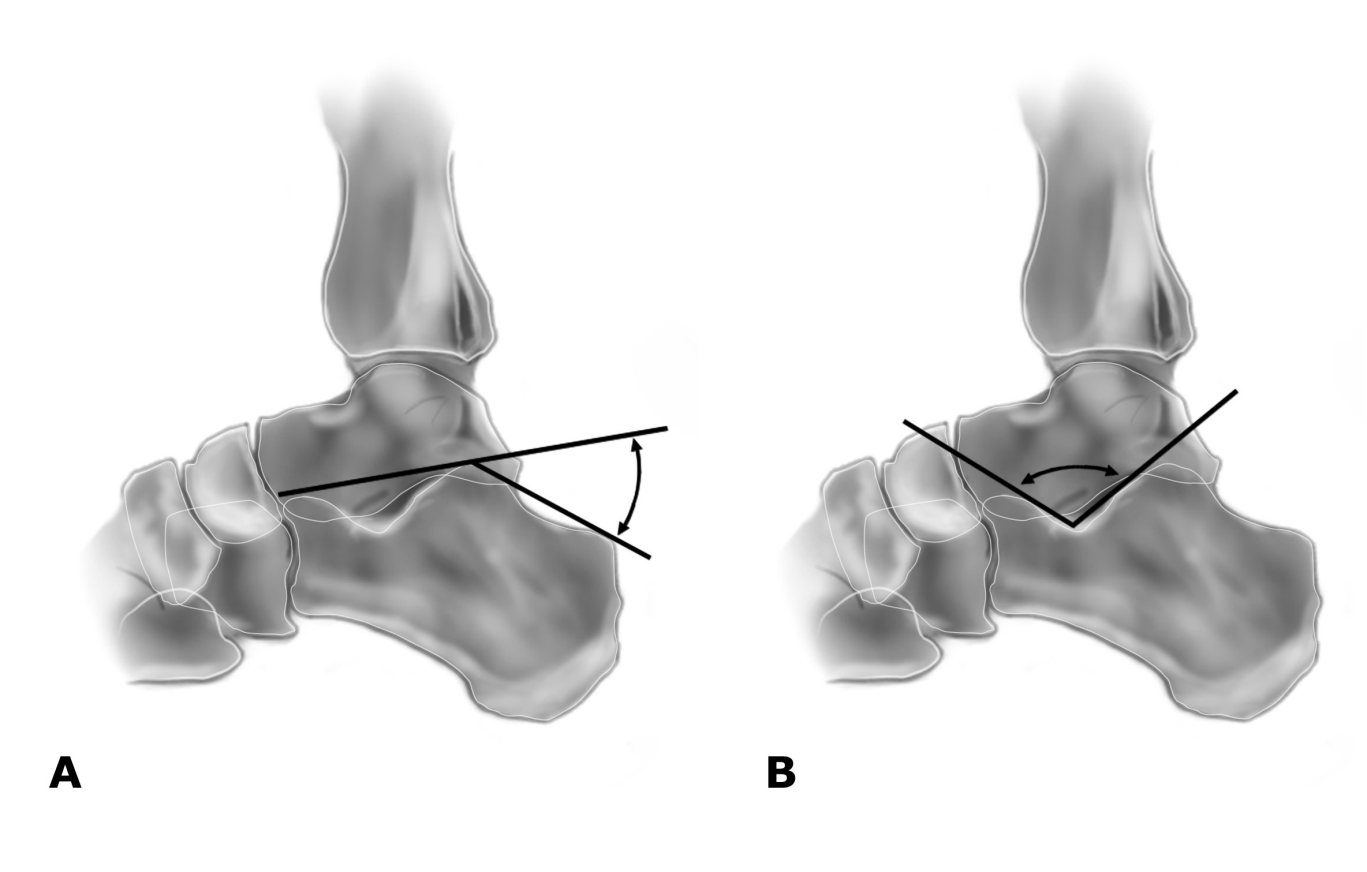
Lateral view of the calcaneus and hindfoot illustrating the measurement techniques for Bohler's angle (A) and the crucial angle of Gissane (B).

Bohler's angle and the crucial angle of Gissane can be measured using plain lateral radiographs of the calcaneus. Bohler's angle is formed by a line drawn from the highest point of the tuberosity to the highest point of the posterior facet and a line drawn from the highest point of the anterior process to the highest point of the posterior facet of the calcaneus (Figure 1A) [1]. The crucial angle of Gissane is seen directly inferior to the lateral process of the talus, formed by a line drawn along the posterior facet of the calcaneus and a line drawn from the anterior process to the sulcus calcaneus (Figure 1B) [1].

It is widely accepted that in the adult population, a normal Bohler's angle [2] is 25 to 40 degrees and a normal crucial angle of Gissane [3] is 100 to 130 degrees. The normal value of these two angles in the paediatric population has not, to our knowledge, been established. The goal of this study was to investigate the normal paediatric values of Bohler's angle and the crucial angle of Gissane.

## Methods

Following ethics committee approval, all plain ankle radiographs in patients from birth to 14 years of age requested by the emergency department at our institution over a five year period from July 2003 to June 2008 were retrospectively evaluated. Patients with plain radiographs showing traumatic, infective or neoplastic bony abnormalities or not displaying a full lateral view of the calcaneus were excluded from the study, leaving 763 patients with plain lateral radiographs of the ankle and calcaneus that had been reported by a radiologist as normal as our study group. Bohler's angle and the crucial angle of Gissane were measured for every patient in the study group using standard digital radiology software. Each measurement was immediately repeated three times by the principal author, and the mean value for each patient was recorded.

As a comparison group, a consecutive series of 100 plain ankle radiographs displaying a full lateral view of the calcaneus in 100 patients aged 30 years to 70 years of age requested by the emergency department at the adult hospital associated with our institution, that had been reported by a radiologist as normal, were retrospectively evaluated. Bohler's angle and the crucial angle of Gissane were measured for every patient in this group using standard digital radiology software. Each measurement was immediately repeated three times by the principal author, and the mean value for each patient was recorded.

For every patient in the study group and in the comparison group, all three repeated measurements were within a two degree range, indicating acceptable intraobserver measure reliability.

The mean Bohler's angle and the mean crucial angle of Gissane in the paediatric study group were compared with the mean values seen in the adult group. Further comparisons were undertaken by dividing the paediatric study group into five smaller groups according to patient age (0-2 years, 3-5 years, 6-8 years, 9-11 years, and 12-14 years of age) and comparing mean values between each group, and between each group and the adult values. Statistical analysis included one-way analysis of variance testing for comparisons between all groups, t-testing with Tukey adjustments for quantification of differences between groups, and a two sample t-test for comparison between overall paediatric and adult values. It should be noted that the crucial angle of Gissane result in the 0-2 year age group did not have equal variance to results seen in the other age groups and therefore multiple two-sample t-tests with Bonferroni adjustments were undertaken when analysing this result. Statistical significance for the crucial angle of Gissane 0-2 year age group comparisons was therefore defined as a p-value of less than 0.01, while for all other comparisons it was defined as a p-value of less than 0.05. For the statistically significant results, 95% confidence intervals were calculated for the difference between the two means.

## Results

In the paediatric study group, the mean Bohler's angle was 35.2 degrees (range 14.3 to 58.1 degrees) and the mean crucial angle of Gissane was 111.3 degrees (range 90.1 to 147 degrees).

In the adult comparison group, the mean Bohler's angle was 39.2 degrees (range 26.2 to 54.9 degrees) and the mean crucial angle of Gissane was 113.8 degrees (range 97.1 to 132 degrees).

The difference in Bohler's angle between the paediatric and adult groups was statistically significant (p < 0.001). The difference in the crucial angle of Gissane between the paediatric and adult groups was also statistically significant (p = 0.004).

**Figure 2:**
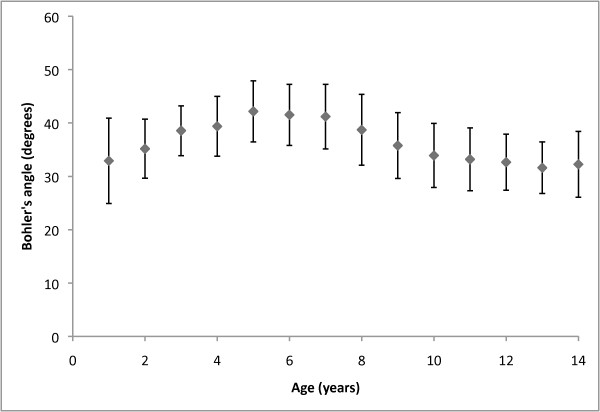
**Distribution of normal Bohler's angle values according to year of age**. Mean values are represented by central diamonds, with surrounding brackets outlining one standard deviation above and below the mean.

**Figure 3:**
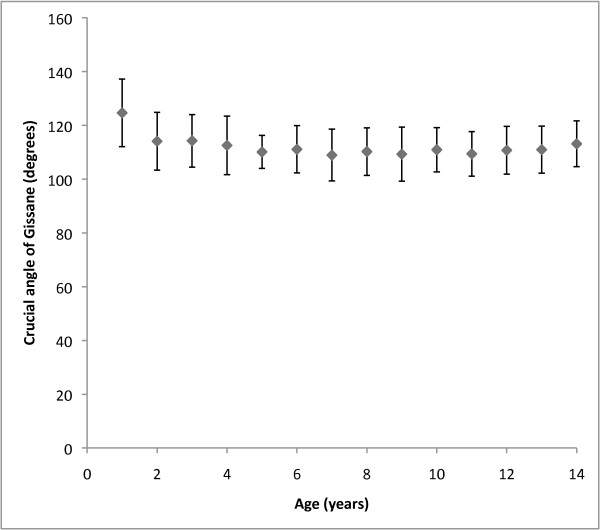
**Distribution of normal crucial angle of Gissane values according to year of age**. Mean values are represented by central diamonds, with surrounding brackets outlining one standard deviation above and below the mean.

Table 1 illustrates the individual group results from our study. Table 2 displays the results of the statistical comparisons performed between the groups in our study. Figure 2 and Figure 3 graphically illustrate our results, with normal paediatric values presented for every year of age.

**Table 1:** Mean values of Bohler's angle and the crucial angle of Gissane for different age groups.

Age (years)	Number of patients	Bohler's Angle (degrees)	Crucial Angle of Gissane (degrees)
**0-2**	59	34.0	119.9

**3-5**	84	40.3	112.0

**6-8**	133	40.5	110.1

**9-11**	219	34.1	110.0

**12-14**	268	32.2	111.0

**0-14**	763	35.2	111.3

**30-70**	100	39.2	113.8

**Table 2:** Statistical comparisons between different age groups of Bohler's angle and the crucial angle of Gissane.

	Bohler's Angle		Crucial angle of Gissane	
**Comparison**	**p-value**	**95% CI**	**p-value**	**95% CI**

A vs. B	<0.001*	[3.50, 9.18]	<0.001*	[3.32, 12.32]

A vs. C	<0.001*	[3.88, 9.12]	<0.001*	[5.45, 14.18]

A vs. D	1		<0.001*	[5.65, 14.15]

A vs. E	0.28		<0.001*	[4.68, 13.11]

A vs. G	<0.001*	[2.43, 7.92]	0.007*	[1.75, 10.45]

B vs. C	1		0.45	

B vs. D	<0.001*	[4.12, 8.41]	0.32	

B vs. E	<0.001*	[6.03, 10.21]	0.85	

B vs. G	0.76		0.65	

C vs. D	<0.001*	[4.59, 8.26]	1	

C vs. E	<0.001*	[6.51, 10.06]	0.85	

C vs. G	0.52		0.009*	[0.62, 6.82]

D vs. E	0.007*	[0.34, 3.38]	0.70	

D vs. G	<0.001*	[3.08, 7.11]	0.002*	[0.98, 6.63]

E vs. G	<0.001*	[5.00, 8.91]	0.043*	[0.06, 5.54]

F vs. G	<0.001*	[2.75, 5.15]	0.004*	[0.79, 4.10]

A: 0-2 years

B: 3-5 years

C: 6-8 years

D: 9-11 years

E: 12-14 years

F: 0-14 years

G: 30-70 years

95% CI: 95% Confidence Interval

*: Statistically significant difference

## Discussion

Bohler's angle and the crucial angle of Gissane are reproducible measures of calcaneal anatomy which may be useful in the management of calcaneal fractures. Our study reports on the normal Bohler's angle and crucial angle of Gissane in a paediatric population. While previous authors have commented on the normal age-related changes in a number of radiographic angles in the paediatric foot [4], to our knowledge this is the first description of the normal values of Bohler's angle and the crucial angle of Gissane in a paediatric population. Our study also shows a statistically significant difference between these paediatric angles and those seen in a normal adult population.

It is interesting to note that in our study Bohler's angle showed several statistically significant differences between smaller age groups within the paediatric population, with interesting age-related variation shown in Figure 2. This is likely due to the asymmetrical ossification pattern of the calcaneus. It has been demonstrated that the primary calcaneal ossification centre begins in the distal two-thirds of the cartilaginous anlage and then proceeds distally and proximally, with the proximal calcaneus and calcaneal part of the subtalar joint ossified last [5]. This asymmetrical progression of primary ossification, in addition to the appearance and fusion of the secondary calcaneal ossification centre, is likely to be responsible for the differences in Bohler's angle seen between age groups in our study.

With one exception, the crucial angle of Gissane showed little variation between age groups in our study. The 0-2 year age group showed a statistically significant increase in this value compared with other groups. This may be due to the asymmetrical ossification pattern of the calcaneus or may be due to difficulties in measuring this angle using radiographs of the very immature calcaneus.

Although we did not separate our results into yearly age groups for statistical analysis to avoid dilution of statistical relevance, it is interesting to note the values of Bohler's angle and the crucial angle of Gissane in the 0-1 year age bracket. While there were only 31 patients in this age bracket, the range of Bohler's angle was 21.7 to 53.5 degrees (mean 32.9 degrees) and the range of the crucial angle of Gissane was 95.5 degrees to 147 degrees (mean 125 degrees). The wide variation seen in this collection of patients highlights the significant radiographic changes that occur during this young age, when only the ossific nucleus is visible.

Bohler's angle following calcaneal fracture has been shown to carry significant prognostic value [6-9]. Loucks and Buckley [6] demonstrated that calcaneal fractures with a markedly diminished Bohler's angle carry a much poorer two-year outcome regardless of treatment. While the differences in Bohler's angle and the crucial angle of Gissane seen between age groups in our study are frequently statistical significant, it is difficult to conclude that these differences reach clinical significance. It is clearly important, however, to have a thorough understanding of the normal anatomy and thus the normal Bohler's angle and crucial angle of Gissane when faced with a calcaneal fracture. We hope that our results will provide helpful normal reference values that orthopaedic surgeons can refer to when assessing the severity of a paediatric calcaneal fracture.

## Conclusions

We have reported on the normal values of Bohler's angle and the crucial angle of Gissane in a paediatric population. We hope that these values will provide a useful comparison to assist with the management of the paediatric calcaneal fracture.

## Competing interests

The authors declare that they have no competing interests.

## Authors' contributions

MB participated in the design of the study, performed the radiological analysis and drafted the manuscript. CW performed the statistical analysis. HC conceived of the study and participated in its design and coordination. All authors read and approved the final manuscript.

## Authors' information

MB is an Orthopaedic Registrar in the Department of Paediatric Orthopaedics at Starship Children's Hospital in Auckland, New Zealand.

CW is a Senior Lecturer in the Department of Engineering Science at The University of Auckland in Auckland, New Zealand.

HC is an Orthopaedic Surgeon in the Department of Paediatric Orthopaedics at Starship Children's Hospital in Auckland, New Zealand.
